# Survey of UK practice for management of breast cancer metastases to the neck

**DOI:** 10.1308/003588412X13171221591736

**Published:** 2012-10

**Authors:** B Bisase, C Kerawala

**Affiliations:** Royal Marsden NHS Foundation Trust,UK

**Keywords:** Current practice, Breast cancer, Neck metastasis, Evidence based

## Abstract

**INTRODUCTION:**

Cervical metastases from breast carcinoma are rare and their management is controversial. Between 1987 and 2002 the American Joint Committee on Cancer (AJCC) staged patients with supraclavicular fossa nodal disease as M1 but the subsequent demonstration that patients with regional stage IV disease had better outcomes than visceral stage IV disease led to a reclassification of the former to stage IIIC in 2003. The literature remains inconsistent regarding the fate of these patients. Despite the attendant morbidity of treatment and lack of knowledge regarding long-term survival, we hypothesised that current practice varies in the UK and a unified approach does not exist. The aim of this study was therefore to determine current practice and opinion of both head and neck specialists and breast cancer clinicians in the UK.

**METHODS:**

Questionnaires were disseminated to 185 head and neck surgeons, breast surgeons and their oncology counterparts. These outlined a clinical scenario of a patient with a history of T3 primary breast cancer presenting with cervical and supraclavicular nodal metastases, with opinion being sought regarding the significance of this status and the individual’s practical approach to the problem. The extent of any proposed neck dissection was also explored.

**RESULTS:**

Of the 117 respondents, a noticeable variation in opinion was evident. Contrary to the current AJCC staging, 61% of clinicians felt that both level V and III metastases represented stage IV disease. There was a tendency towards aggressive surgical treatment with a third recommending comprehensive neck dissection despite a lack of evidence base. A disparity was noted between adjuvant treatments offered and the final pN stage.

**CONCLUSIONS:**

This study suggests that at present there is widespread inconsistency in the management of breast carcinoma cervical metastases in the UK. There is a need to unify practice with an evidence base in order to improve informed multidisciplinary decision making and, ultimately, patient care. This study goes some way to supporting multicentre collaboration in order to achieve that aim.

From 1982 to 2002 supraclavicular lymph node metastases from breast cancer were considered metastatic and conferred stage IV disease.^1^ This classification of stage was justified by evidence suggesting that the prognosis in patients with neck disease was poor, with five-year survival rates in the order of 5–34%.^2–4^ Evidence also suggested this poor prognosis to be similar to that of patients with metastases in other sites (ie bone, liver)^5^ with treatment not seeming to influence survival.^6^ Since limited literature exists guiding treatment for isolated ipsilateral cervical metastases, management remains controversial. This could be explained by the low occurrence of neck metastases in breast carcinoma (around 1%)^3,7^ and by the scarce evidence from randomised trials that establish prognosis and optimal treatment.

The demonstration that patients with regional stage IV disease had better outcomes than visceral stage IV disease led to a reclassification in 2003. This revision classifies metastasis to the supraclavicular lymph node (SCLN) as N3c/pN3c. A new stage (stage IIIC) was introduced and includes any T stage with N3 disease (pN3a, pN3b, pN3c).^2,7^ As patients with distant metastases were considered incurable, many with neck disease only received palliative care. This treatment approach became controversial after a study by Brito *et al* involving 70 patients with SCLN positive breast cancer who received aggressive treatment that included induction chemotherapy, surgery, post-operative chemotherapy and irradiation.^8^ At a median follow-up time of 8.5 years, the disease free survival and overall survival of these patients was equivalent to those with stage IIIB without distant metastasis and significantly better than stage IV patients. As such, classifying SCLN as a distant metastasis may lead to undertreatment of patients.

Metastases elsewhere in the jugular chain are not mentioned in AJCC staging. Known to head and neck (H&N) surgeons as Robbins level V, the SCLN region is the region bordered by the clavicle, the posterior border of the sternocleidomastoid and the anterior border of the trapezius. The jugular chain, however, known to H&N surgeons as Robbins levels II, III and IV, is the region deep to the sternocleidomastoid from the mastoid down to the clavicle and bordered superiorly by the posterior belly of the digastric and anteriorly by the hyosternal strap muscles.

In a study by Sesterhenn *et al* reporting just 12 cases, it was suggested that jugular chain metastasis may occur in as many as 50%.^9^ Where the incidence of the SCLN metastasis recurrence is reported at around 1% of patients with breast cancer history, one may extrapolate 0.5% for the jugular chain. Even with larger studies such as that by Pedersen *et al* analysing 45,854 breast cancer cases and 305 cases with SCLN disease over a 26-year period, upper jugular chain involvement as opposed to supraclavicular fossa only (SCLN) was not discussed.^3^ The question therefore remains whether these represent distant metastases when compared with SCLN (supraclavicular fossa only) metastasis.

There is emerging evidence that tumour receptor status may change dynamically during the natural history of the disease and influence the management of recurrence accordingly. Schuler *et al* believed that the loss of steroid receptor expression contributed to tumour resistance to endocrine therapy.^10^ Amir discussed results of the largest pooled analysis of two prospective studies assessing receptor status in patients with recurrent breast cancer.^11^ The findings demonstrated a change in receptor status in the order of 5–34%. Reliable published concrete evidence regarding the frequency and relevance of change in receptor status is scarce, possibly leaving clinicians uncertain of how to manage this information appropriately.

Evidence regarding adjuvant or multimodality treatment approaches varies and, as such, clinicians or multidisciplinary teams (MDTs) may struggle to best advise on management strategies. Marcial reported that adjuvant prophylactic post-operative primary site irradiation reduced locoregional (including neck) recurrences to less than 10%.^12^ He went on to suggest that with therapeutic irradiation, tumour control rates of at least 50% were possible in cases of locoregional cervical recurrence after mastectomy. Control rates were best with irradiation after surgery that removed the gross bulk of recurrent neck disease and, in reference to isolated SCLN, there was even better control with higher doses of radiation when combined with surgery. Brito *et al* recommended combined modality treatment involving chemotherapy, surgery and radiotherapy in order to effect a better prognosis for ipsilateral isolated SCLN.^8^

Various H&N surgeons and oncologists are likely to be exposed to this type of referral (direct or via MDT) at some stage in their practice albeit rarely (SCLN metastasis 1%).^3,7^ Since the literature is scarce and inconsistent regarding the fate of breast carcinoma patients with cervical disease, our impression was that in view of the attendant morbidity of treatment and lack of knowledge regarding the influence of management on long-term survival, a unified approach to these patients does not exist.^6^ We therefore hypothesised that current practice varies across the UK.

This may affect the management of patients adversely if suboptimal practices are advised by local MDTs or, indeed, if no unified policies or guidelines exist. If we were to demonstrate such a variation in practice, we would hope this would carry weight to support multicentre collaboration in data collection and encourage consensus groups to reach evidence-based guidelines. It was hence the aim of this study to establish current practice and opinions among H&N and breast surgeons and their oncology counterparts in the UK.

## Methods

**Figure 1:**
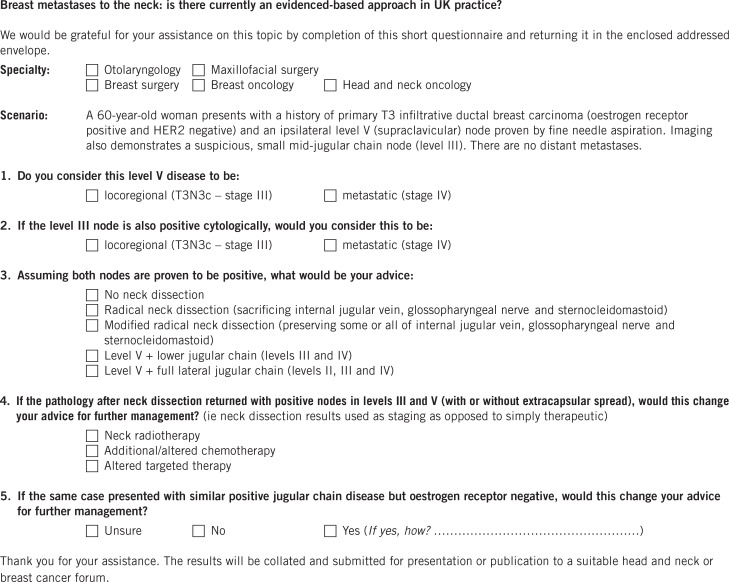
Consultant surgeon questionnaire

Overall, 185 questionnaires were disseminated to consultant H&N surgeons from otolaryngology, oral and maxillofacial surgery (OMFS) as well as to breast surgeons and their oncology counterparts (Fig 1). These individuals were identified around the UK using regional cancer centre data from NHS trust internet sites in conjunction with H&N and breast oncology associations’ information. Care was taken to ensure that the search covered all regions across the UK in order to minimise any potential geographical bias.

With the aid of a clinical scenario, a questionnaire was designed to identify information including the respondents’ specialty, their opinion on the significance of supraclavicular and other cervical metastases from breast carcinoma, and their practical approach to the clinical problem including types of neck dissection (ND) (selective vs comprehensive) where appropriate. The questionnaire also explored the surgeons’ opinions on receptor status and preferences for adjuvant treatments. The scenario used was that of a 60-year-old woman with a history of primary T3 infiltrative ductal breast carcinoma (oestrogen receptor [ER] positive and HER2 negative) presenting with an ipsilateral level V node proven by fine needle aspiration. Concurrent imaging also demonstrated a suspicious mid-jugular chain level III node but no distant metastases.

## Results

**Figure 2:**
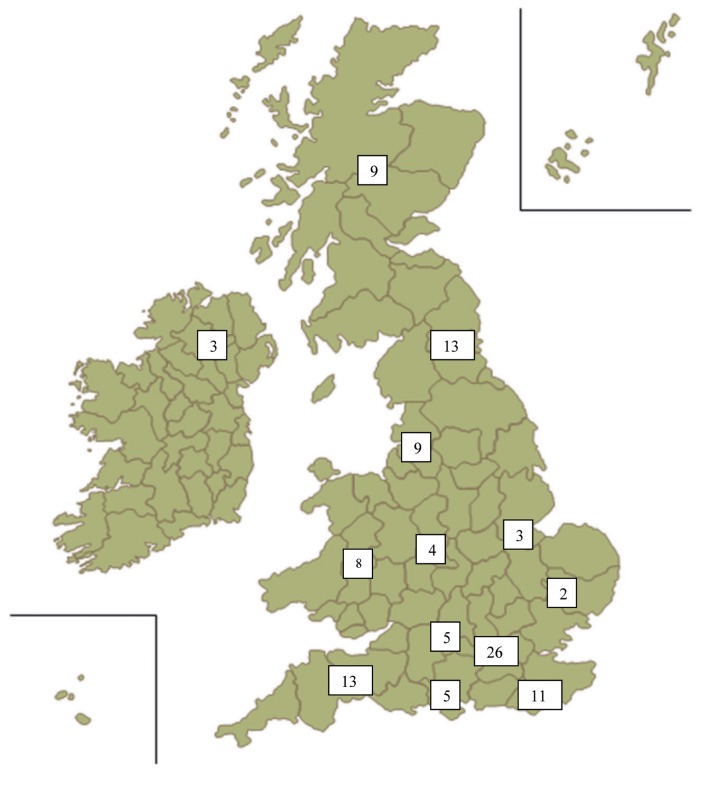
The distribution of respondents across the UK

**Figure 3:**
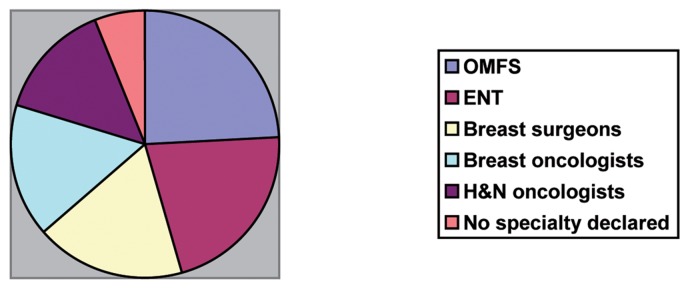
Respondents’ clinical specialties

There were 122 responses (66%), for which the respondent’s region was not identifiable in 11. Of all the responses, 5 were incomplete, leaving a working total of 117. The distribution across the UK is demonstrated in Figure 2. The variation between specialties in respondents was minimal with a marginal majority of the surgeons practising OMFS (Fig 3).

In contrast to the current AJCC staging, 71 clinicians (61%, 71/117) felt both level III and V metastases represented stage IV disease. Of these, 38 were H&N surgeons and 33 breast surgeons or oncologists. Regarding the significance of upper jugular chain nodes compared with supraclavicular nodes, 30/117 (26%) considered a difference in staging between level III (distant metastasis) and level V (locoregional). Of this group, 67% (20/30) were breast surgeons or oncologists.

**Figure 4:**
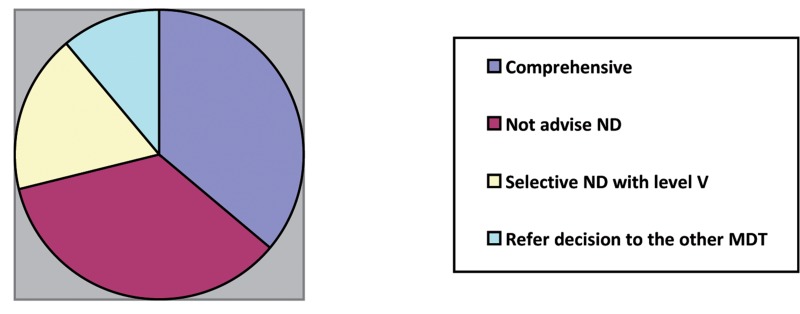
Summary of the cohorts’ opinions regarding neck dissection

When opinions were explored regarding further management of the neck in the event that both nodes were proven to be metastatic, 41 clinicians (35%) did not support surgery. When questioned about surgical management of the neck, the responses regarding what extent of ND would be suggested are summarised in Figure 4. Radical ND, whether modified or not, were recommended by 42/117 clinicians (36%) overall.

The distribution of the types of ND preferred between clinicians’ specialties is demonstrated in Table 1. This revealed a tendency towards modified radical ND in the clinical scenario.

**Table 1:** The distribution of the types of neck dissection preferred between clinicians’ specialties

	Otolaryngology	Oral and maxillofacial surgery	Head and neck oncology	Breast surgery	Breast oncology	Total
No neck dissection	4	2	4	17	14	**41**
Radical neck dissection	8	2	4	0	0	**14**
Modified radical neck dissection	9	9	8	1	1	**28**
Lower jugular chain and level V	1	2	0	2	1	**6**
Full jugular chain and level V	5	5	3	0	2	**15**
None of the above or ‘ask the other multidisciplinary team’	3	5	1	1	3	**13**

Thirteen clinicians did not comment on the type of ND they advised but felt that it should be the decision of the opposing MDT, with 9/13 H&N clinicians suggesting the decision would best lie with breast surgeons or oncologists and 4/13 breast clinicians commenting that the type of surgery would be better decided by H&N surgeons.

Where clinicians were questioned on their preference of further management in the event of pathology after ND demonstrating positive nodes in levels III and V, neck radiotherapy (36%) followed by additional/altered chemotherapy (33%) were most popular. Just under a quarter (23%) did not feel this information would change their advice regarding additional treatment. Although 33 clinicians were unsure as to the change in management if the patient was ER negative, 43 were confident it would not change their advice. Of these, 21 were breast oncologists.

## Discussion

Responses revealed a noticeable variation in understanding, opinions and practice. Although infrequent, involvement of otolaryngology/OMFS/H&N oncologists in the management of such patients may occur for a number of reasons including: discussion of any positive cancer cytology for a neck lump; aiding diagnosis when inconclusive cytology requires histology and excision is necessary; and if the agreed practice by both MDTs is for ND or radiotherapy for local control in isolation or in combination as a part of multimodal strategies.

The available literature does not necessarily make it easier to prevent these inconsistencies. For example, Debois and others demonstrated that patients presenting with supraclavicular metastases have a poor prognosis and that more than 50% succumb to disease within three years of diagnosis.^6,13,14^ In contrast, Olivotto *et al* showed that a significant minority (approximately 13%) may be long-term survivors.^7^ In their study, breast cancer specific survival for patients with supraclavicular disease at diagnosis was intermediate between survival of patients presenting with IIIB and M_1_ (other) disease through the first decade of follow-up. Between 10 and 20 years after diagnosis, the overall and breast cancer specific survival rates of the supraclavicular and IIIB patients converge and remain significantly better than cases presenting with M_1_ disease. It must be noted, however, that in this study the majority of patients with supraclavicular metastases had the diagnosis made clinically and not cytohistologically. Such cases were only included if review of the initial oncology consultation made a clear description of a hard mass, consistent with metastases in the supraclavicular region. As such, it is possible that patients without supraclavicular metastases could have been misclassified and so apparent survival might have been improved.

In support of downstaging SCLN metastases, Brito *et al* demonstrated that at a median follow-up time of 8.5 years, the disease free survival and overall survival seen in these patients was equivalent to that seen in stage IIIB patients without distant metastasis and significantly better than that seen in stage IV patients.^8^ With such inconsistencies in the literature, it is not surprising that in our study, in contrast to current guidelines and AJCC staging, two-thirds of clinicians felt both levels III and V metastases represented stage IV disease.

There was a tendency towards aggressive surgical treatment in some of the groups approached in this study despite a lack of evidence base in addition to a disparity between adjuvant treatments offered and the final pN stage. Of note is the number of clinicians that suggested aggressive surgical neck management such as modified radical ND or level II–V dissection (43/117). There is very limited evidence regarding the true benefit of jugular chain ND on local recurrence, overall or disease specific survival. The majority of those suggesting comprehensive ND (*n*=63) were H&N clinicians (88%, 56/63) with the remaining 12% being breast clinicians.

This difference may be due to the lack of evidence on the best surgical approach in the literature and, despite attending morbidities, H&N clinicians defaulting to what they know in the context of managing locally advanced squamous cell carcinoma. While Sesterhenn *et al* reported neck node metastases of breast cancer located superiorly to the supraclavicular region in more than 50% of cases, their study only included 12 patients.^9^ Whether lymph node metastases situated above the supraclavicular region represents distant metastases remains an important unanswered question. As demonstrated by our respondents (26% considered a difference in staging between levels V [locoregional] and III [distant metastasis]), this nodal presentation may warrant clarification in the current AJCC staging system.

The attending risk of surgical morbidity and evidence that multimodal local and systemic therapy yielded better outcomes (76–87% complete remission) may explain the 41/117 respondents who did not advocate ND.^3,7^ The majority of these were breast clinicians (85%, 35/41). It must be noted that the evidence supporting systemic therapy alone in the absence of surgery yielding a better outcome (as suggested by these 41 respondents) remains to be proven and is not readily identifiable in the current literature.^3,12,15^ Indeed, Pedersen *et al* went on to demonstrate a 43% complete remission in patients who did not have surgery but who had radiotherapy with/without systemic therapy alone.^3^ One must bear in mind that although their study presents data on one of the largest cohorts (*n*=305), it was limited by its non-randomised nature and up-to-date systemic treatments were not used (study period 1977–2003).

Owing to a short, focused questionnaire, we were not able to explore loss of receptor status in this study. The significance of this is that some believe that, secondary to long-term endocrine hormone therapy, a reduction in ER expression occurs. There is emerging evidence that tumour receptor status may change dynamically during the natural history of the disease and may therefore influence the management of recurrence. Schuler *et al* believed that the loss of steroid receptor expression contributes to tumour resistance to endocrine therapy.^10^ However, the context of their belief was on a background of only three case reports on patients presenting with breast cancer metastasis to the head and neck.

Amir discussed results of the largest pooled analysis of two prospective studies assessing receptor status in patients with recurrent breast cancer.^11^ Biopsies were prospectively obtained and analysed in both studies. Receptor status discordance between primary and recurrent disease occurred in 12.6% of patients for ER, in 34.1% for progesterone receptor and in 5.4% for HER2. Gain or loss of receptor expression was similar for ER and HER2 but progesterone receptor loss was more frequent than gain (76% vs 8%). Other concrete evidence regarding the relevance of the change in receptor status remains scarce.

The purpose of questioning clinicians on their preference of further management in the event of pathology after ND demonstrating positive nodes in levels III and V was to gauge whether clinicians used ND results for staging the patient and therefore offered further therapy on the basis of this finding. This was in contrast to offering multimodal therapy at the outset or approaching ND as a procedure for local control only. In this study, neck radiotherapy (36/117) followed by additional/altered chemotherapy (33/117) were popular. As alluded to previously, remission (either complete or partial) and prognosis in general is better in cases managed by combined adjuvant therapies.^3,12^

## Conclusions

The findings of this study suggest a widespread inconsistency in the management of breast carcinoma patients presenting with cervical disease in the UK. This occurs on the background of limited reliable randomised data. A greater appreciation of current differences of opinions and interpretation of cervical metastasis between both MDTs (H&N and breast clinicians) may be helpful. The disparity of opinions may reflect the limited exposure clinicians have to such cervical metastases. This study demonstrates a variation in practice that we hope carries weight to support multicentre collaboration in data collection and to encourage consensus groups to reach unified evidence-based guidelines. In turn, we would expect this to help avoid suboptimal management of patients in this cohort for whom unified policies or guidelines do not exist.
